# Pathogens That Cause Acute Febrile Illness Among Children and Adolescents in Burkina Faso, Madagascar, and Sudan

**DOI:** 10.1093/cid/ciab289

**Published:** 2021-04-02

**Authors:** Florian Marks, Jie Liu, Abdramane Bassiahi Soura, Nagla Gasmelseed, Darwin J Operario, Brian Grundy, John Wieser, Jean Gratz, Christian G Meyer, Justin Im, Jacqueline Kyungah Lim, Vera von Kalckreuth, Ligia Maria Cruz Espinoza, Frank Konings, Hyon Jin Jeon, Raphaël Rakotozandrindrainy, Jixian Zhang, Ursula Panzner, Eric Houpt

**Affiliations:** 1 International Vaccine Institute, Seoul, Republic of Korea; 2 University of Antananarivo, Antananarivo, Madagascar; 3 Cambridge Institute of Therapeutic Immunology and Infectious Disease, University of Cambridge School of Clinical Medicine, Cambridge Biomedical Campus, Cambridge, United Kingdom; 4 Division of Infectious Diseases and International Health, Department of Medicine, University of Virginia, Charlottesville, Virginia, USA; 5 Institut Supérieur des Sciences de la Population, University of Ouagadougou, Burkina Faso; 6 Faculty of Medicine, University of Gezira, Wad Medani, Sudan; 7 Faculty of Medicine, Duy Tan University, Da Nang, Vietnam; 8 Institute of Tropical Medicine, Eberhard Karls University, Tübingen, Germany; 9 Swiss Tropical and Public Health Institute, Basel, Switzerland; 10 University of Basel, Basel, Switzerland

**Keywords:** febrile illness, Africa/sub-Saharan Africa, whole blood, TaqMan array

## Abstract

**Background:**

The etiology and optimal clinical management of acute febrile illness (AFI) is poorly understood.

**Methods:**

Blood samples taken from study participants with acute fever (≥37.5°C) or a history of fever and recruited into the previous Typhoid Fever Surveillance in Africa (TSAP) study were evaluated using a polymerase chain reaction (PCR)-based TaqMan-Array Card designed to detect a panel of bacterial, viral, and parasitic pathogens. Clinical metadata were also assessed.

**Results:**

A total of 615 blood samples available for analysis originated from Burkina Faso (n = 53), Madagascar (n = 364), and Sudan (n = 198) and were taken from participants ranging in age from 0–19 years. Through the TaqMan-Array Card, at least 1 pathogen was detected in 62% (33 of 53), 24% (86 of 364), and 60% (118 of 198) of specimens from Burkina Faso, Madagascar, and Sudan, respectively. The leading identified pathogen overall was *Plasmodium* spp*.,* accounting for 47% (25 of 53), 2.2% (8 of 364), and 45% (90 of 198) of AFI at the respective sites. In Madagascar, dengue virus was the most prevalent pathogen (10.2%). Overall, 69% (357 of 516) of patients with clinical diagnoses of malaria, respiratory infection, or gastrointestinal infection were prescribed a World Health Organization guideline-recommended empiric antibiotic, whereas only 45% (106 of 237) of patients with pathogens detected were treated with an antibiotic exerting likely activity.

**Conclusions:**

A PCR approach for identifying multiple bacterial, viral, and parasitic pathogens in whole blood unveiled a diversity of previously undetected pathogens in AFI cases and carries implications for the appropriate management of this common syndrome.

## Teaser

Children and adolescents with acute febrile illness at 3 African sites underwent testing for multiple bacterial, viral, and parasitic pathogens in whole blood, revealing a diversity of pathogens with important implications for appropriate management of this common syndrome.

In African and other low- and middle-income countries, up to 80% of children who visit a healthcare facility present with an acute febrile illness (AFI) [1–3]. AFI is a broad clinical syndrome, generally considered to be caused by an infectious agent and characterized by fever with or without accompanying symptoms. AFI-related research is challenging for several reasons. First, there is no conventional case definition for AFI [4], as is available for diarrhea and pneumonia, rendering reliable global burden estimates of AFI difficult [4–6]. AFI studies have used varying definitions of subjective and objective fever, including different temperature cutoffs, fever duration, and requirements for accompanying clinical indicators in diverse patient populations [7]. Second, there are myriad infectious agents and noninfectious causes that trigger fever. While AFI cases in Africa are often attributed to malaria or viral infections [8–11], dozens of bacterial, fungal, and other parasitic causes exist [12]. Therefore, for an accurate diagnosis, a wide range of tests must be applied. Due to practical and economic constraints, most studies focus on a narrow range of suspect pathogens [13].

The Typhoid Fever Surveillance in Africa Program (TSAP) was a multisite fever surveillance study conducted prospectively in 10 sub-Saharan African countries [2]. The project’s main goal was to identify *Salmonella* Typhi and invasive nontyphoidal *Salmonella* through blood culture. In this study, we investigate the presence of pathogens in whole blood specimens collected from children and adolescents enrolled in the TSAP study using a multiple polymerase chain reaction (PCR) approach with the goal of obtaining additional insights into the etiology of AFI.

## METHODS

### Study Participants

We analyzed venous blood samples collected at the time of enrollment into TSAP between 2011 and 2013 for which sufficient volume was still available; samples had been maintained at –80°C and were shipped on dry ice for testing in 2018. Samples were available from Burkina Faso, Sudan, and Madagascar [1, 14], and we focused on samples from children and adolescents. Details of the TSAP study have been published previously [2]. Briefly, recruitment healthcare facilities were public institutions providing general medical care to patients of all ages. Inclusion criteria were residence in the study area and fever [2]. The purpose of TSAP was focused on the detection of typhoid fever by blood culture. Clinical examination, clinical diagnoses, other laboratory testing such as malaria smear, and management practices were not altered by the study protocol and reflected local routine practice. Ethical clearance for TSAP, which included additional pathogen identification performed through this work, was obtained from committees of the International Vaccine Institute in South Korea, the Comité d’Ethique pour la Recherche en Santé of the Ministry of Health in Burkina Faso, the Comité d’Ethique of the Ministry of Health of the Republic of Madagascar, and the National Research Ethics Review Committee of the National Ministry of Health in Sudan. Additional approval was obtained for this work by the University of Virginia Institutional Review Board.

### TaqMan Array Card

The PCR-based TaqMan Array Card (TAC) system [15–18] used in this analysis was designed to detect AFI-associated viral, bacterial, parasitic, and fungal pathogens. The TAC system allows simultaneous detection of a wide range of pathogens (see Supplementary Table 1). Details and assay specifications of the TAC system are described elsewhere [17]. TAC is a 384-well real-time PCR-based microfluidic system for simultaneous detection of nucleic acid templates. We designed primers and probes for the detection of 50 pathogens (19 viruses, 25 bacteria, 3 fungi, and 3 parasites) recognized to cause AFI [12]. Primers and probes were used at concentrations of 900 nM and 250 nM, respectively. The assay required 800 μL of whole blood from which total nucleic acid was extracted using the High Pure Viral Nucleic Acid Large Volume Kit (Roche, Basel, Switzerland). Cards were loaded with 75 μL nucleic acid extract and 25 μL TaqMan Fast Virus 1-Step Master Mix (Life Technologies, Thermo Fisher Scientific, Carlsbad, CA). Phocine herpesvirus 1 (10^6^ copies) and MS2 bacteriophage (10^7^ copies) were added to monitor extraction and amplification efficiency. One extraction blank control was included for each batch to exclude contamination [19, 20]. No template and positive controls were used [19–21]. Cards were run on the ViiA7 Real-time PCR System (Life Technologies, Thermo Fisher Scientific, Carlsbad, CA) as follows: 1 reverse transcription cycle for 10 minutes at 50°C, 1 denaturation cycle for 20 seconds at 95°C, and 40 two-step PCR and data detection cycles of 3 seconds at 95°C and 30 seconds at 60°C. A sample was considered positive if a reaction yielded quantification cycles (Cq) of <35 or Cq<40 if confirmable by sequencing (110 positive samples were sequence-confirmed).

### Statistical Analyses

Analyses were performed using STATA statistical package (StataCorp LLC, version 14, College Station, TX) and SPSS version 26. When evaluating hemoglobin values, the site-specific altitude was used to adjust the values according to World Health Organization (WHO) guidelines [14, 22, 23]. Age- and sex-stratified *z* scores for height for age were generated using WHO growth standards [24, 25]. Body temperature calculated by axillary measurement only was normalized by adding 0.4°C for comparability with tympanic measurements [26]. Odds ratios (ORs) and confidence intervals for the presence or absence of symptoms were calculated for each individual pathogen with binary logistic regression models controlling for age as an ordinal covariate and gender and the 3 clinical sites as nominal covariates. Two-sided *P* values <.05 were considered statistically significant.

## RESULTS

We tested 615 blood samples from individuals who presented with AFI; 8.6% (53 of 615) of patients from Burkina Faso, 59.2% (364 of 615) from Madagascar, and 32.2% (198 of 615) from Sudan. Study participants ranged in age from 0 to 19 years. Clinical characteristics of the participants are listed in Table 1. The majority (86%, 531 of 615) were aged between 11 and 19 years and presented as outpatients with acute fever of ≤3 days duration. Overall, 10% (60 of 615) and 7% (41 of 615) of participants had moderate or severe stunting, respectively. Common symptoms that accompanied fever included headache, 77% (471 of 615); cough, 39% (238 of 615); sore throat, 35% (216 of 615); vomiting, 24% (145 of 615); abdominal pain, 22% (136 of 615); and diarrhea, 16% (99 of 615). The leading clinical diagnoses in Burkina Faso and Sudan were malaria, 60% (32 of 53) and 63% (125 of 198), and respiratory tract infections, 17% (9 of 53) and 20% (39 of 198), respectively. In Madagascar, the most frequent clinical diagnosis was respiratory tract infection, 64% (234 of 364), followed by gastrointestinal infection, 15% (54 of 364). A wide range of antibiotics were prescribed (Table 1).

**Table 1.** Baseline Clinical Characteristics of Study Participants Included in the Analysis Overall and by Site

Characteristic	Burkina Faso (n = 53)		Madagascar (n = 364)		Sudan (n = 198)		Overall^a^ (n = 615)	
	**n**	**%**	**n**	**%**	**n**	**%**	**n**	**%**
Age, years								
1–10	29	(55)	24	(7)	31	(16)	84	(14)
11–19	24	(45)	340	(93)	167	(84)	531	(86)
Median (range)	9 (1–19)		15 (1–19)		13 (2–19)		14 (1–19)	
Gender								
Male	23	(43)	137	(41)	91	(48)	251	(43)
Female	30	(57)	200	(59)	98	(52)	328	(57)
Body temperature, °C								
≤39.0	46	(87)	350	(96)	113	(59)	509	(84)
>39.0	7	(13)	14	(4)	77	(41)	98	(16)
Median (range)	39.0 (38.0–41.4)		38.4 (37.0–40.5)		39.0 (38.0–40.6)		38.4 (37.0–41.4)	
Fever duration, days								
≤3	47	(89)	318	(87)	183	(95)	548	(90)
>3	6	(11)	46	(13)	9	(5)	61	(10)
Body mass index for age, *z* score								
Above –2	38	(72)	252	(75)	155	(99)	445	(82)
–2 to –3 (moderate)	5	(9)	53	(16)	2	(1)	60	(11)
Below –3 (severe)	10	(19)	31	(9)	0	(0)	41	(7)
Hemoglobin^b^								
Normal	19	(41)	90	(60)	70	(49)	179	(53)
Mild anemia	12	(26)	14	(9)	40	(28)	66	(19)
Moderate anemia	15	(33)	7	(5)	31	(22)	53	(16)
Severe anemia	0	(0)	39	(26)	2	(1)	41	(12)
Clinical signs^c^								
Abdominal pain	8	(15)	58	(16)	70	(35)	136	(22)
Cough	13	(25)	148	(41)	77	(38)	238	(39)
Diarrhea	9	(17)	71	(20)	19	(19)	99	(16)
Headache	29	(55)	277	(76)	165	(83)	471	(77)
Rash	0	(0)	12	(3)	5	(3)	17	(3)
Sore throat	0	(0)	133	(37)	83	(42)	216	(35)
Vomiting	17	(32)	58	(16)	70	(19)	145	(24)
None of the above	7	(13)	20	(5)	7	(4)	34	(6)
Primary clinical diagnosis								
Respiratory tract infection	9	(17)	234	(64)	39	(20)	282	(46)
Urinary tract infection	0	(0)	4	(1)	2	(1)	6	(1)
Gastrointestinal tract infection	5	(9)	54	(15)	7	(4)	66	(11)
Malaria	32	(60)	11	(3)	125	(63)	168	(27)
Other infections	5	(9)	33	(9)	10	(5)	48	(8)
Other^d^	2	(4)	28	(8)	15	(8)	45	(7)
Antibiotic treatment at discharge^c^								
Ampicillin	0	(0)	71	(20)	26	(13)	97	(16)
Amoxicillin	5	(9)	123	(34)	37	(19)	165	(27)
Ceftriaxone	4	(8)	3	(1)	1	(0.5)	8	(1)
Chloramphenicol	0	(0)	34	(9)	1	(0.5)	35	(6)
Ciprofloxacin	5	(9)	21	(6)	5	(3)	31	(5)
Gentamicin	2	(4)	112	(31)	3	(2)	117	(19)
Macrolides	0	(0)	2	(1)	16	(8)	18	(3)
Tetracyclines	0	(0)	10	(3)	0	(0)	10	(2)
Trimethoprim/Sulfamethoxazole	17	(32)	25	(7)	1	(0.5)	43	(7)
Antimalarial	9	(17)	10	(3)	114	(58)	133	(22)

^a^ In case of missing data, not all data points sum to 615.

^b^ Adjusted for participant age and altitude.

^c^ Patients can have multiple clinical signs and have multiple antibiotic treatments.

^d^ Included trauma, allergy, asthma, insect bite, and rheumatism.

We assessed the subset of individuals tested in this project against those published in the TSAP study. Participants of our subset were, in comparison, older, had a lower temperature, and had less anemia than the others in the TSAP (Supplementary Table 2 for full details).

Overall, the TAC system identified at least 1 pathogen in 39% (237 of 615) of the specimens evaluated (Table 2). In total, 22 pathogens were found, including 14 bacterial species, 4 viruses, 2 fungi, and 2 parasites. The most frequent pathogen with 20% (123 of 615) identified overall was *Plasmodium* (47%, 25 of 53 in Burkina Faso; 2.2%, 8 of 364 in Madagascar; and 45%, 90 of 198 in Sudan). Further species-specific assays showed that 92% were *P. falciparum,* 2% were mixed infections, and 6% were unspeciated. At the Madagascar sites, dengue virus was recovered from 10.2% (37 of 364) of samples, followed by cytomegalovirus (CMV) with 5.5% (20 of 364), *Bartonella* with 2.5% (9 of 364), and *Plasmodium* spp. with 2.2% (8 of 364). At the Sudanese site, at least 1 pathogen was identified in 60% (118 of 198) of samples; these included dengue virus, *Candida* spp., *Coxiella burnetii*, *Escherichia coli*, *Klebsiella pneumoniae*, *Aeromonas* spp., and *Staphylococcus aureus* (Table 2). There was 1 case of *Rickettsia* spp. and 1 case of *Mycobacterium tuberculosis*. At the Burkina Faso site, leading identified pathogens included *Plasmodium* in 47% (25 of 53), CMV in 15% (8 of 53), dengue virus in 11% (6 of 53), and *E. coli* in 4% (2 of 53). Four *Bartonella*-positive samples from Madagascar were able to be further speciated by sequencing to be *Bartonella quintana*. At all sites, *Schistosoma mansoni* was found.

**Table 2.** Pathogens Detected in Whole Blood EDTA Samples Using TaqMan Array Card During Acute Febrile Illness Among Study Participants from Burkina Faso, Madagascar, and Sudan

	Burkina Faso, 53 AFI Cases	Madagascar, 364 AFI Cases	Sudan, 198 AFI Cases
Bacteria	Crude Cases (%)	Crude Cases (%)	Crude Cases (%)
*Acinetobacter baumannii*	nd	nd	2 (1.0)
*Aeromonas* spp.	nd	1 (0.3)	3 (1.5)
*Bartonella* spp.	1 (2)	9 (2.5)	nd
*Coxiella burnetii*	nd	5 (1.4)	4 (2.0)
*Escherichia coli*	2 (4)	5 (1.4)	9 (4.5)
*Klebsiella oxytoca*	nd	1 (0.3)	1 (0.5)
*Klebsiella pneumoniae*	1 (2)	1 (0.3)	6 (3.0)
*Mycobacterium tuberculosis*	nd	nd	1 (0.5)
*Neisseria meningitidis*	1 (2)	nd	nd
*Pseudomonas aeruginosa*	nd	1 (0.3)	nd
*Rickettsia* spp.	1 (2)	nd	1 (0.5)
*Staphylococcus aureus*	nd	2 (0.5)	4 (2.0)
*Streptococcus pneumoniae*	nd	1 (0.3)	3 (1.5)
*Salmonella*	nd	2 (0.5)	nd
Viruses			
Dengue	6 (11)	37 (10.2)	14 (7.1)
Enterovirus	nd	2 (0.5)	nd
Rift Valley fever	nd	1 (0.3)	nd
Cytomegalovirus	8 (15)	20 (5.5)	6 (3.0)
Fungi			
*Candida* spp.	nd	5 (1.4)	6 (3.0)
*Histoplasma* spp.	nd	nd	1 (0.5)
Parasites			
*Plasmodium* spp.	25 (47)	8 (2.2)	90 (45)
*Schistosoma* spp.	1 (2)	4 (1.1)	3 (1.5)
Samples with pathogen found^a^	33 (62)	86 (24)	118 (60)
Samples with no pathogen found	20 (38)	278 (76)	80 (40)

Abbreviations: AFI, acute febrile illness; nd, not detected.

^a^ Samples could have multiple coinfections detected.

**Figure 1.**
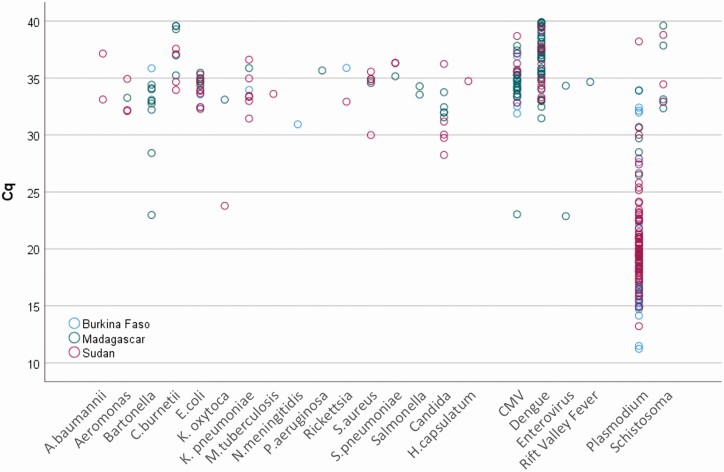
Quantities of pathogens detected in blood using the TaqMan Array Card. Quantification cycle (Cq) values, an inverse metric of pathogen load, are shown on the *y*- axis for the different pathogens detected. In this study, the Cq values of most bacterial, fungal, and viral targets ranged from 30 to 40, with a few exceptions of early Cq values such as *Bartonella* spp., *Klebsiella oxytoca*, and Enterovirus spp. Therefore, most of these samples had low pathogen loads. Alternatively, low Cq-values could have resulted from sample degradation, in particular, for viral pathogens with RNA genomes, as the whole blood samples used for this retrospective testing had been stored for several years. The Cq-values of *Plasmodium* spp., on the other hand, showed a much wider dynamic range. Abbreviation: CMV, cytomegalovirus.

Multiple pathogens were identified in 51 samples (22% of positive samples). Two pathogens were found in 16% (38 of 237) of specimens, 3 pathogens in 5% (12 of 237), and 5 pathogens in 1 patient, 0.4% (1 of 237). *Plasmodium falciparum* was the most common coinfecting pathogen (36 of 51 coinfections), followed by dengue virus (19 of 51 coinfections) and CMV (15 of 51 coinfections). Malaria smears were reported positive in 98 specimens, among which TAC detected *Plasmodium* in 70% (69 of 98). *Plasmodium* quantitative PCR quantities were higher in these smear-positive (Cq = 19.4 ± 4.1) vs smear-negative detections (Cq = 22.0 ± 5.2, n = 54, *P* < .050). The quantities of pathogens were not appreciably different between countries (Figure 1).

### Correlation Between Clinical Features and Pathogens Identified

We sought to determine how the clinical presentation and management of patients correlated with the pathogens that were identified. The respective associations are shown in Table 3. While data for many pathogens were sparse, we noted the following: *Candida* spp. was associated with abdominal pain (adjusted OR [aOR], 4.3; *P* = .03) and constipation (aOR, 7.1; *P* = .03). *Klebsiella pneumoniae* was positively associated with cough (aOR, 12.1; *P* = .02), while *Plasmodium* spp. was negatively associated with cough (aOR, 0.6; *P* = .04; Table 3). Dengue was associated with the absence of localized symptoms (aOR, 3.15; *P* = .02).

**Table 3.** Association of Clinical Features of Overall Study Participants With Specific Pathogens Detected Using TaqMan Array Card

Pathogen	Symptom	Adjusted Odds Ratio^a^	95% Confidence Interval	*P* Value
*Candida* spp.	Abdominal pain	4.3	1.1–16	.03
*Candida* spp.	Constipation	7.1	1.3–40	.03
Cytomegalovirus	Diarrhea	2.1	.9–4.8	.08
Dengue	Cough	0.5	.3–1.0	.06
Dengue	No symptom	3.2	1.2–8.6	.02
*Escherichia coli*	Cough	2.6	.9–7.4	.08
*Klebsiella pneumoniae*	Cough	12.1	1.5–101	.02
*Plasmodium*	Cough	0.6	.35–.98	.04

^a^All patient symptoms (8 total), as shown in Table 1, were analyzed for associations with all pathogens with more than 8 detections (9 total) and when no pathogen was detected. Odds ratios (ORs) were adjusted for age as an ordinal covariate, gender, and the 3 clinical sites as nominal covariates. In the interest of space, only ORs with a *P* value of <.10 are shown.

We then examined the clinical diagnoses and their relationships to the pathogens, the only significant association was between clinical malaria and the detection of *Plasmodium* spp. Specifically, 52% (87 of 168) of clinical malaria diagnoses had *Plasmodium* spp. detected, whereas among other clinical diagnoses, only in 8% (36 of 447) was *Plasmodium* spp. identified (aOR, 2.73; *P < *.01). Furthermore, the *Plasmodium* spp. quantity was greater when malaria was the primary clinical diagnosis (Cq = 19.9 ± 4.1 vs 22.22 ± 5.9 when detected among other primary clinical diagnoses; *P* = .03).

We retrospectively determined whether the antibiotics prescribed empirically were in line with the guidelines for the particular clinical diagnosis or for the pathogen detected. Overall, in 69% (357 of 516) of patients with a primary clinical diagnosis of malaria, respiratory tract infection, or gastrointestinal infection, an antibiotic aligned with WHO guidance was prescribed [27]. By contrast, 45% (106 of 237) of the participants were prescribed antibiotics that, given the benefit of hindsight, were inappropriate with regard to the specific pathogen(s) detected (see Supplementary Table 3 for a list of pathogens and active therapies). The likelihood of prescribing an appropriate antibiotic for a pathogen increased if a recommended antibiotic for the clinical diagnosis was prescribed (55% vs 24% if a recommended antibiotic was not prescribed for the clinical diagnosis; aOR, 3.2; 95% CI 1.8–5.9; *P* < .01); however, both of these rates were quite low. We also assessed malaria. From the 168 clinically diagnosed malaria cases, 123 were confirmed to harbor *Plasmodium* spp. parasites; 61% of them (75 of 123) received an artemisinin-based or quinine therapy, as is appropriate.

## Discussion

This study provides an important epidemiologic characterization of AFI in 3 sub-Saharan African countries to complement previous work [13, 28, 29]. The study involved predominantly outpatient children and adolescents from sites in Burkina Faso, Madagascar, and Sudan. While blood cultures revealed less than 1% positivity rate in these samples, our TAC approach identified 22 pathogens in 38.5% (237 of 615) of specimens, including bacteria in 9%. These molecular detection rates probably still underestimate the true extent of infections because of the small quantity of blood available [18].

Findings from the Sudanese site contrasted with results from the original TSAP study, whereby a pathogen could be detected by blood culture in only 2% (16 of 644) [1]. One hypothesis was that the extremely hot climate compromised blood culture performance by diminishing bacterial growth [30]. Our present molecular data, by contrast, indicated the presence of several bacterial pathogens in 17.2% (34 of 198), including *E. coli*, *K. pneumoniae*, *S. aureus*, *Aeromonas*, and *Coxiella*. In Burkina Faso and Sudan, *Plasmodium* was the prevailing pathogen, followed by dengue virus and *Candida*. An important finding was substantial dengue prevalence at all 3 sites, even though dengue is understudied in Madagascar, with only 1 report from 2006 [31]. Future data are needed for decision-making on various control measures, including application of dengue vaccines. Another finding of public health importance was the detection of the Rift Valley fever virus and a meningococcal case that occurred after the meningococcal A vaccine was introduced in Burkina Faso. The relative frequency of severe anemia in Madagascar constitutes an important finding for further investigation, particularly given the absence of malaria in the highlands.

There were several limitations to this study. First, we could not assess disease outcomes, so we could not determine the benefit of particular clinical diagnoses or specific therapies. We noted a wide range of antibiotics prescribed. Sometimes these choices were not in line with WHO recommendations for the respective disease. This practice of antibiotic treatment that is not in line with recognized guidelines is common and exists in all countries, rich and poor [32, 33]. The reasons for the clinical diagnosis-recommended therapy mismatches in this study were not probed as it was not the subject of this study, and the prescribed choices may be acceptable. Our purpose in assessing clinical diagnosis–therapy alignment was to compare it to pathogen diagnosis–therapy alignment. We found clinical diagnosis–therapy mismatches in 31%, while pathogen diagnosis-appropriate therapy mismatches were seen in 55%, which is considerably higher. We do not overinterpret these numbers given that the clinical diagnosis and treatment data were collected observationally under routine conditions; it merely suggests substantial room to improve pathogen-specific diagnosis and therapy.

There were examples of both inactive antibiotics (eg, undertreatment of common bacterial pathogens such as *E. coli*, *K. pneumoniae*, and *S. aureus*) and unnecessary antibiotics (eg, antimalarial treatment when no *Plasmodium* was detected by PCR). For instance, of 40 cases of dengue monoinfection, 88% received antibiotics. Whether improved pathogen-specific diagnosis and management can improve outcomes is an important area for future investigation.

A second limitation was that there were no blood specimens from clinically healthy patients to serve as controls. This is particularly an issue for *Plasmodium* spp., as asymptomatic parasitemia without disease may occur frequently. A quantitative case definition of a few thousand parasites per microliter has been used frequently to enhance clinical specificity [34–36], which corresponds to a Cq-value of approximately 21 with these assays. Our finding that clinical malaria cases and smear-positive cases had higher quantities of *Plasmodium* parasites fits this notion. Applying this cutoff, the prevalence of *Plasmodium* in these 3 countries would drop to 32%, 1%, and 30% for the 3 sites. How frequently asymptomatic nucleic acid detection in blood occurs for other pathogens is less clear. Certainly, CMV detection is often not sufficient evidence for clinical disease. Beyond *Plasmodium* and CMV, PCR is a gold-standard clinical diagnostic for most of the pathogens in this study, such as dengue virus, *Bartonella*, *Coxiella*, and *Rickettsia* [37–39]. For culturable bacterial pathogens, the performance of direct PCR on blood vs blood culture has shown a substantial rate of blood culture–negative/PCR-positive assays [40], as was the case in this study. We believe this reflects stochastic detection of the inherently low quantity of pathogens in blood, and we speculate that either culture or PCR-positive results may be meaningful, having demonstrated this in septic patients with blood culture–negative/PCR-positive specimens in Uganda [18]. Therefore, we surmise that in this clinical setting, most nucleic acid detections in cases of AFI should be considered important unless proven otherwise. That said, we recognize that results from larger PCR studies with control blood specimens constitute an important gap in our knowledge.

A third limitation was that we could only test specimens in small amounts of residual blood conserved from the underlying TSAP study. This skewed the population age to older children compared with the parent study. It also could have enriched for less sick individuals. However, in general, these were outpatient individuals and thus not severely ill. Nonetheless, our findings are likely most generalizable to the adolescent population. Finally, since malaria smears were performed under routine conditions and not under a research protocol, we expect this explains some of the malaria smear–PCR mismatch.

The optimal clinical management of AFI is uncertain. WHO guidelines recommend assessing fever for malaria with rapid antigen or smear testing, with empiric treatment if positive. If there are respiratory symptoms, antibiotics for pneumonia should be considered. If bloody diarrhea exists, antibiotic treatment to cover *Shigella* and *Campylobacter* should be pursued. Of course clinical judgment is complex and does not always align with guidelines [41]. For all of these reasons, data on the optimal management of AFI, particularly in sub-Saharan Africa, is limited and needs further consideration.

## Conclusions

In summary, this TAC study leveraged systematic AFI surveillance to provide a granular picture of prevailing pathogens. This work showed a substantial pathogen diversity missed by blood culture and should be considered when conducting future assessments of disease burden. The impact of these molecular detections and implications on management require further study.

## Supplementary Data

Supplementary materials are available at *Clinical Infectious Diseases online*. Consisting of data provided by the authors to benefit the reader, the posted materials are not copyedited and are the sole responsibility of the authors, so questions or comments should be addressed to the corresponding author.

ciab289_suppl_Supplementary_Table_S1

ciab289_suppl_Supplementary_Table_S2

ciab289_suppl_Supplementary_Table_S3

## Notes

***Author contributions.*** F. M., F. K., V. v K., A. B. S., R. R., U. P., and N. G. conceived the underlying Typhoid Fever Surveillance in Africa (TSAP) study. E. H., J. L., J. W., and J. G. developed the TaqMan Array Card assays. A. B. S., N. G., V. v K., R. R., L. M. C. E., J. I., and F. K. coordinated the TSAP study in the 3 countries. U. P., J. L., D. O., J. Z., J. W., J. G., and E. H. conducted laboratory analyses. U. P., E. H., and B. G. conducted the scientific and statistical analyses. U. P., E. H., and F. M. wrote the manuscript. C. G. M., H. J. J., J. I., and J. K. L. critically reviewed the manuscript. All authors provided critical feedback and helped shape the research, analysis and manuscript.

***Financial support.*** This work was supported by the National Institutes of Health (National Institute of Health; K24AI102972 to E. H.). B. G. is supported by the National Institute of Health (T32 AI007046). The Bill and Melinda Gates Foundation provided financial support for the TSAP study (OPP1127988).

***Potential conflicts of interest.*** All authors: No reported conflicts of interest. All authors have submitted the ICMJE Form for Disclosure of Potential Conflicts of Interest. Conflicts that the editors consider relevant to the content of the manuscript have been disclosed.

## References

[1] Marks F , von Kalckreuth V, Aaby P, et al. Incidence of invasive salmonella disease in sub-Saharan Africa: a multicentre population-based surveillance study. Lancet Glob Health 2017; 5:e310–23. 28193398 10.1016/S2214-109X(17)30022-0 PMC5316558

[2] von Kalckreuth V , Konings F, Aaby P, et al. The typhoid fever surveillance in Africa Program (TSAP): clinical, diagnostic, and epidemiological methodologies. Clin Infect Dis 2016; 62 Suppl 1:S9–S16. 26933028 10.1093/cid/civ693 PMC4772831

[3] Herlihy JM , D’Acremont V, Hay Burgess DC, Hamer DH. Diagnosis and treatment of the febrile child. In: Black RE, Laxminarayan R, Temmerman M, Walker N, eds. Reproductive, maternal, newborn, and child health: disease control priorities, 3rd ed. Vol 2. Washington (DC): The International Bank for Reconstruction and Development / The World Bank; 2016.

[4] Crump JA . Time for a comprehensive approach to the syndrome of fever in the tropics. Trans R Soc Trop Med Hyg 2014; 108:61–2. 24463580 10.1093/trstmh/trt120 PMC3916746

[5] Crump JA , Kirk MD. Estimating the burden of febrile illnesses. PLoS Negl Trop Dis 2015; 9:e0004040. 26633014 10.1371/journal.pntd.0004040 PMC4668833

[6] Global Burden of Disease 2017 Disease and Injury Incidence and Prevalence Collaborators. Global, regional, and national incidence, prevalence, and years lived with disability for 354 diseases and injuries for 195 countries and territories, 1990–2017: a systematic analysis for the Global Burden of Disease Study 2017. Lancet 2018; 392(10159):1789–858.

[7] Prasad N , Sharples KJ, Murdoch DR, Crump JA. Community prevalence of fever and relationship with malaria among infants and children in low-resource areas. Am J Trop Med Hyg 2015; 93:178–80. 25918207 10.4269/ajtmh.14-0646 PMC4497891

[8] Animut A , Mekonnen Y, Shimelis D, Ephraim E. Febrile illnesses of different etiology among outpatients in four health centers in Northwestern Ethiopia. Jpn J Infect Dis 2009; 62:107–10. 19305049

[9] Crump JA , Morrissey AB, Nicholson WL, et al. Etiology of severe non-malaria febrile illness in northern Tanzania: a prospective cohort study. PLoS Negl Trop Dis 2013; 7:e2324. 23875053 10.1371/journal.pntd.0002324 PMC3715424

[10] D’Acremont V , Kilowoko M, Kyungu E, et al. Beyond malaria— causes of fever in outpatient Tanzanian children. N Engl J Med 2014; 370:809–17. 10.1056/NEJMoa1214482 24571753

[11] Njama-Meya D , Clark TD, Nzarubara B, Staedke S, Kamya MR, Dorsey G. Treatment of malaria restricted to laboratory-confirmed cases: a prospective cohort study in Ugandan children. Malar J 2007; 6:7. 17239256 10.1186/1475-2875-6-7 PMC1797179

[12] Iroh Tam PY , Obaro SK, Storch G. Challenges in the etiology and diagnosis of acute febrile illness in children in low- and middle-income countries. J Pediatric Infect Dis Soc 2016; 5:190–205. 27059657 10.1093/jpids/piw016 PMC7107506

[13] Rhee C , Kharod GA, Schaad N, et al. Global knowledge gaps in acute febrile illness etiologic investigations: a scoping review. PLoS Negl Trop Dis 2019; 13:e0007792. 31730635 10.1371/journal.pntd.0007792 PMC6881070

[14] Panzner U , Pak GD, Aaby P, et al. Utilization of healthcare in the Typhoid Fever Surveillance in Africa Program. Clin Infect Dis 2016; 62 Suppl 1:S56–68. 26933023 10.1093/cid/civ891 PMC4772834

[15] Abade A , Eidex RB, Maro A, et al. Use of TaqMan Array Cards to screen outbreak specimens for causes of febrile illness in Tanzania. Am J Trop Med Hyg 2018; 98:1640–2. 29611511 10.4269/ajtmh.18-0071 PMC6086183

[16] Hercik C , Cosmas L, Mogeni OD, et al. A combined syndromic approach to examine viral, bacterial, and parasitic agents among febrile patients: a pilot study in Kilombero, Tanzania. Am J Trop Med Hyg 2018; 98:625–32. 29280432 10.4269/ajtmh.17-0421 PMC5929188

[17] Liu J , Ochieng C, Wiersma S, et al. Development of a TaqMan array card for acute-febrile-illness outbreak investigation and surveillance of emerging pathogens, including Ebola virus. J Clin Microbiol 2016; 54:49–58. 26491176 10.1128/JCM.02257-15 PMC4702733

[18] Moore CC , Jacob ST, Banura P, et al. Etiology of sepsis in Uganda using a quantitative polymerase chain reaction-based TaqMan Array Card. Clin Infect Dis 2019; 68:266–72. 29868873 10.1093/cid/ciy472 PMC6321855

[19] Liu J , Gratz J, Amour C, et al. A laboratory-developed TaqMan Array Card for simultaneous detection of 19 enteropathogens. J Clin Microbiol 2013; 51:472–80. 23175269 10.1128/JCM.02658-12 PMC3553916

[20] Liu J , Gratz J, Amour C, et al. Optimization of quantitative PCR methods for enteropathogen detection. PLoS One 2016; 11:e0158199. 27336160 10.1371/journal.pone.0158199 PMC4918952

[21] Kodani M , Winchell JM. Engineered combined-positive-control template for real-time reverse transcription-PCR in multiple-pathogen-detection assays. J Clin Microbiol 2012; 50:1057–60. 22170926 10.1128/JCM.05987-11 PMC3295119

[22] World Atlas. Where is Ouagadougou, Burkina Faso. October 19, 2015. Available at: https://www.worldatlas.com/af/bf/ce/where-is-ouagadougou.html.

[23] World Health Organization. The global prevalence of anemia in 2011. 2015. Available at: https://apps.who.int/iris/bitstream/handle/10665/177094/9789241564960_eng.pdf;jsessionid=5186C8EF5E807A1FD61DC99E2730DC93?sequence=1.

[24] World Health Organization. WHO child growth standards. November 11, 2006. Available at. https://www.who.int/childgrowth/standards/technical_report/en/.

[25] World Health Organization. Growth reference 5–19 years. 2007. Available at: https://www.who.int/growthref/en/.

[26] Oyakhirome S , Profanter K, Kremsner PG. Assessment of fever in African children: implication for malaria trials. Am J Trop Med Hyg 2010; 82:215–8. 20133994 10.4269/ajtmh.2010.09-0419 PMC2813159

[27] World Health Organization. WHO recommendations on child health: guidelines approved by the WHO Guidelines Review Committee. May 2, 2017 Available at: https://www.who.int/publications/i/item/WHO-MCA-17.08. 40455892

[28] Elven J , Dahal P, Ashley EA, et al. Non-malarial febrile illness: a systematic review of published aetiological studies and case reports from Africa, 1980-2015. BMC Med 2020; 18:279. 32951596 10.1186/s12916-020-01744-1 PMC7504660

[29] Maze MJ , Bassat Q, Feasey NA, Mandomando I, Musicha P, Crump JA. The epidemiology of febrile illness in sub-Saharan Africa: implications for diagnosis and management. Clin Microbiol Infect 2018; 24:808–14. 29454844 10.1016/j.cmi.2018.02.011 PMC6057815

[30] Ombelet S , Barbé B, Affolabi D, et al. Best practices of blood cultures in low- and middle-income countries. Front Med (Lausanne) 2019; 6:131. 31275940 10.3389/fmed.2019.00131 PMC6591475

[31] Ratsitorahina M , Harisoa J, Ratovonjato J, et al. Outbreak of dengue and chikungunya fevers, Toamasina, Madagascar, 2006. Emerg Infect Dis 2008; 14:1135–7. 18598641 10.3201/eid1407.071521 PMC2600361

[32] Centers for Disease Control and Prevention. Antibiotic use in the United States, 2018 update: progress and opportunities. Atlanta, GA: US Department of Health and Human Services, CDC; 2019.

[33] Rogawski ET , Platts-Mills JA, Seidman JC, et al. Use of antibiotics in children younger than two years in eight countries: a prospective cohort study. Bull World Health Organ 2017; 95:49–61. 28053364 10.2471/BLT.16.176123 PMC5180352

[34] Tabue RN , Njeambosay BA, Zeukeng F, et al. Case definitions of clinical malaria in children from three health districts in the north region of Cameroon. Biomed Res Int 2019; 2019:9709013. 31139663 10.1155/2019/9709013 PMC6500661

[35] Neafsey DE , Juraska M, Bedford T, et al. Genetic diversity and protective efficacy of the RTS,S/AS01 malaria vaccine. N Engl J Med 2015; 373:2025–37. 26488565 10.1056/NEJMoa1505819 PMC4762279

[36] Marks F , Evans J, Meyer CG, et al. High prevalence of markers for sulfadoxine and pyrimethamine resistance in *Plasmodium falciparum* in the absence of drug pressure in the Ashanti region of Ghana. Antimicrob Agents Chemother 2005; 49:1101–5. 15728909 10.1128/AAC.49.3.1101-1105.2005 PMC549270

[37] Zeaiter Z , Fournier PE, Greub G, Raoult D. Diagnosis of *Bartonella* endocarditis by a real-time nested PCR assay using serum. J Clin Microbiol 2003; 41:919–25. 12624010 10.1128/JCM.41.3.919-925.2003 PMC150267

[38] Fournier PE , Raoult D. Comparison of PCR and serology assays for early diagnosis of acute Q fever. J Clin Microbiol 2003; 41:5094–8. 14605144 10.1128/JCM.41.11.5094-5098.2003 PMC262519

[39] Paris DH , Dumler JS. State of the art of diagnosis of rickettsial diseases: the use of blood specimens for diagnosis of scrub typhus, spotted fever group rickettsiosis, and murine typhus. Curr Opin Infect Dis 2016; 29:433–9. 27429138 10.1097/QCO.0000000000000298 PMC5029442

[40] Dark P , Blackwood B, Gates S, et al. Accuracy of LightCycler® SeptiFast for the detection and identification of pathogens in the blood of patients with suspected sepsis: a systematic review and meta-analysis. Intensive Care Med 2015; 41:21–33. 25416643 10.1007/s00134-014-3553-8

[41] World Health Organization. WHO informal consultation on fever management in peripheral health care settings: a global review of evidence and practice. January 2013. Available at: https://www.who.int/tdr/publications/year/2013/fever-management/en/.

