# Supplementary material for: Pathogens That Cause Acute Febrile Illness Among Children and Adolescents in Burkina Faso, Madagascar, and Sudan
Source: Clin Infect Dis. 2021 Apr 2;73(8):1338–45. doi: 10.1093/cid/ciab289 (PMC8528393; doi:10.1093/cid/ciab289)
Supplement: ciab289_suppl_Supplementary_Table_S1 [file ciab289_suppl_supplementary_table_s1.docx]

| **Classification** | **Pathogen** | **Gene target** |
| --- | --- | --- |
| **Viruses** | Bundibugyo virus | VP40 |
|  | Crimean–Congo hemorrhagic fever (CCHF) virus | NP |
|  | Chikungunya virus | NSP4 |
|  | Cytomegalovirus (CMV) | gB & IE2 exon 5 |
|  | Dengue virus | 3’NC |
|  | Dengue virus 1 | NS5 |
|  | Dengue virus 2 | E |
|  | Dengue virus 3 | prM |
|  | Dengue virus 4 | prM |
|  | Ebola virus | NP |
|  | Enterovirus | 5'UTR |
|  | Hepatitis E virus | ORF3 |
|  | Human Immunodeficiency Virus I (HIV I) | LTR |
|  | HIV II | LTR |
|  | Herpes simplex Virus 1 (HSV 1) | gB |
|  | HSV 2 | gB |
|  | Lassa virus |  |
|  | Marburg virus | VP40 |
|  | Monkeypox virus |  |
|  | O'nyong'nyong Virus (ONNV) | E1 |
|  | Rift Valley Fever Virus | L |
|  | Sudan virus | NP |
|  | West Nile Virus | 3'NC |
|  | Yellow Fever Virus | RdRp |
|  | Zika virus |  |
| **Bacteria** | *Acinetobacter baumannii* | bla_OXA-51_ |
|  | Aeromonas spp. | Aerolysin |
|  | Bartonella spp. | ssrA |
|  | Brucella spp. | IS711 |
|  | *Coxiella burnetii* | IS1111 |
|  | *Escherichia coli*/Shigella spp. | uidA |
|  | *Enterococcus faecalis* | ddl |
|  | *Haemophilus influenzae* | bexA |
|  | *Klebsiella oxytoca* | pehX |
|  | *Klebsiella pneumoniae* | Diguanylate cyclase |
|  | Leptospira spp. | LipL32 |
|  | Mycobacterium avium complex (MAC) | ITS |
|  | *Mycobacterium tuberculosis* | IS6110 |
|  | *Neisseria meningitidis* | sodC |
|  | *Orientia tsutsugamushi* | 47KD |
|  | *Pseudomonas aeruginosa* | gyrB |
|  | Rickettsia spp. | 23S |
|  | *Staphylococcus aureus* | Glutamate synthase & sodA |
|  | *Streptococcus agalactiae* | cfb |
|  | *Streptococcus pneumonia* | lytA |
|  | *Streptococcus pyogenes* |  |
|  | *Salmonella enterica* | ttr |
|  | *Salmonella enterica* Typhi | STY0201 |
|  | *Yersinia pestis* | caf1 |
| **Fungi** | Candida spp. | 18S |
|  | *Cryptococcus neoformans* | ITS2 |
|  | *Histoplasma capsulatum* | ITS1 |
| **Parasites** | Plasmodium spp. | 18S |
|  | *Plasmodium falciparum* | 18S |
|  | *Plasmodium knowlesi* | 18S |
|  | *Plasmodium ovale* | 18S |
|  | *Plasmodium vivax* | 18S |
|  | *Plasmodium malariae* | 18S |
|  | Schistosoma spp. | ITS |
|  | *Toxoplasma gondii* | 18S |

**Supplemental Table 1.** Gene targets for pathogen detection by TaqMan Array
