# Supplementary material for: Pathogens That Cause Acute Febrile Illness Among Children and Adolescents in Burkina Faso, Madagascar, and Sudan
Source: Clin Infect Dis. 2021 Apr 2;73(8):1338–45. doi: 10.1093/cid/ciab289 (PMC8528393; doi:10.1093/cid/ciab289)
Supplement: ciab289_suppl_Supplementary_Table_S2 [file ciab289_suppl_supplementary_table_s2.docx]

**Supplemental Table 2**: Baseline group characteristics of participants included in this analysis *versus* the remaining TSAP 0-19 year old population. All patients with available blood were included.

| **Characteristics** |  | **TAC tested**  **(n=615, all sites)** | | **Not tested**  **(n=2230, all sites)** | ***p*-value** |  |
| --- | --- | --- | --- | --- | --- | --- |
| **Age** |  | N (%) | | N(%) |  |  |
|  | 1-10 years | 84 (14) | | 1723 (77) | <0.001 |  |
|  | 11-19 years | 531(86) | | 507 (23) |  |  |
| **Gender** |  |  | |  |  |  |
|  | Male | 251 (43) | | 1103 (49) | 0.009 |  |
|  | Female | 328 (57) | | 1127 (51) |  |  |
| **Body temperature** |  |  |  |  |  |  |
|  | ≤39.0 °C | 509 (84) | | 685 (31) | <0.001 |  |
|  | >39.0 °C | 98 (16) | | 1545 (69) |  |  |
| **Fever duration** |  |  |  |  |  |  |
|  | ≤3 days | 548 (90) | | 2041 (92) | 0.234 |  |
|  | >3 days | 61 (10) | | 189 (8) |  | |
| **Clinical severity** |  |  |  |  |  |  |
|  | Inpatient/Admission | 4 (1) | | 53 (2) | 0.008 |  |
|  | Outpatient | 603 (99) | | 2177 (98) |  | |
| **BMI-for-age z-score** |  |  |  |  |  |  |
|  | Above -2 | 445 (82) | | 1519 (81) | 0.92 |  |
|  | -2 to -3 (moderate) | 60 (11) | | 170 (9) | 0.187 |  |
|  | Below -3 (severe) | 41 (7) | | 179 (10) | 0.139 |  |
| **Hemoglobin^b^** |  |  |  |  |  |  |
|  | Normal | 179 (53) | | 697 (47) | 0.041 |  |
|  | Mild anemia | 66 (19) | | 351 (24) | 0.11 | |
|  | Moderate anemia | 53 (16) | | 412 (28) | <0.001 |  |
|  | Severe anemia | 41 (12) | | 33 (2) | <0.001 |  |
| **Clinical signs^c^** |  |  |  |  |  |  |
|  | Abdominal pain | 136 (22) | | 355 (16) | <0.001 |  |
|  | Cough | 238 (39) | | 979 (44) | 0.021 | |
|  | Diarrhea | 99 (16) | | 431 (19) | 0.069 |  |
|  | Headache | 471 (77) | | 1227 (55) | <0.001 |  |
|  | Rash | 17 (3) | | 50 (2) | 0.447 |  |
|  | Sore throat | 216 (35) | | 356 (16) | <0.001 |  |
|  | Vomiting | 145 (24) | | 706 (32) | <0.001 |  |
|  | Other | 204 (33) | | 559 (25) | <0.001 |  |
| **Primary clinical diagnosis** |  |  |  |  |  |  |
|  | Respiratory tract infection | 282 (46) | | 939 (37) | <0.001 |  |
|  | Urinary tract infection | 6 (1) | | 7 (0) | 0.014 | |
|  | Gastrointestinal tract infection | 66 (11) | | 505 (20) | <0.001 |  |
|  | Malaria | 168 (27) | | 881 (34) | <0.001 |  |
|  | Other infections | 48 (8) | | 155 (6) | 0.112 |  |
|  | Other^e^ | 45 (7) | | 70 (3) | <0.001 |  |

^a^In case of missing data, not all data points sum to 615

^b^adjusted for participant age and altitude

^c^patients can have multiple clinical signs

^d^included trauma, allergy, asthma, insect bite, rheumatism
