# Supplementary material for: Pathogens That Cause Acute Febrile Illness Among Children and Adolescents in Burkina Faso, Madagascar, and Sudan
Source: Clin Infect Dis. 2021 Apr 2;73(8):1338–45. doi: 10.1093/cid/ciab289 (PMC8528393; doi:10.1093/cid/ciab289)
Supplement: ciab289_suppl_Supplementary_Table_S3 [file ciab289_suppl_supplementary_table_s3.docx]

**Supplemental Table 3:** Diagnoses and recommended or appropriate therapies

| **Clinical diagnosis** | **Recommended therapy*** | **Prescribed # (%)** |
| --- | --- | --- |
| Malaria clinical diagnosis | Artemesinin-based combination therapies, Quinine | 137/168 (82%) |
| Respiratory infection diagnosis | Beta lactams, Chloramphenicol, Macrolides | 190/282 (67%) |
| GI infection diagnosis | Ceftriaxone, Ciprofloxacin, Macrolides, TMP-SMZ | 30/66 (45%) |
| Total clinical diagnoses |  | 357/516 (69%) |

*Recommended therapy based on WHO recommendations on child health: guidelines approved by the WHO Guidelines Review Committee. Geneva: World Health Organization; 2017 <https://www.who.int/publications/i/item/WHO-MCA-17.08> (Accessed on August 26, 2020)

| **Pathogen diagnosis** | **Likely appropriate therapy**** | **Prescribed # (%)** |
| --- | --- | --- |
| *A. baumannii* | Carbapenem | 0/2 (0%) |
| Aeromonas | Ceftriaxone, Ciprofloxacin, Chloramphenicol, Tetracyclines, Gentamicin, TMP-SMZ | 1/4 (25%) |
| Bartonella | Gentamicin, Macrolides, Tetracyclines, TMP-SMZ | 4/10 (40%) |
| *C. burnetii* | Ciprofloxacin, Macrolides, Tetracyclines, TMP-SMZ | 1/9 (11%) |
| Candida | Fluconazole | 0/11 (0%) |
| CMV | No antibiotic | 8/34 (24%) |
| Dengue | No antibiotic | 6/57 (11%) |
| E. coli | Chloramphenicol, Ciprofloxacin, Beta lactams, Gentamicin, TMP-SMZ | 8/16 (50%) |
| Enterovirus | No antibiotic | 1/2 (50%) |
| Histoplasma | Antifungal* (none documented in study) | 0/1 (0%) |
| *K. oxytoca* | Any cephalosporin, Ciprofloxacin, Gentamicin, TMP-SMZ | 1/2 (50%) |
| *K. pneumoniae* | Any cephalosporin, Ciprofloxacin, Gentamicin, TMP-SMZ | 6/8 (75%) |
| *M. tuberculosis* | RIPE* (none documented in study) | 0/1 (0%) |
| *N. meningitidis* | Ampicillin, Amoxicillin, Ceftriaxone, Ciprofloxacin, Chloramphenicol, Macrolides, TMP-SMZ, Tetracyclines | 1/1 (100%) |
| *P. aeruginosa* | Ciprofloxacin | 0/1 (0%) |
| *Plasmodium* spp. | ACT therapies, Quinine | 75/123 (61%) |
| *Rickettsia* | Chloramphenicol, Tetracyclines | 0/2 (0%) |
| Rift Valley Fever | No antibiotic | 0/1 (0%) |
| *S. aureus* | Any cephalosporin, Tetracyclines, TMP-SMZ | 2/6 (33%) |
| *S. pneumoniae* | Ampicillin, Amoxicillin, Any cephalosporin, Macrolides | 0/4 (0%) |
| *Salmonella* spp. | Ampicillin, Amoxicillin, Ceftriaxone, Chloramphenicol, Ciprofloxacin, Macrolides, TMP-SMZ, | 2/2 (100%) |
| *Schistosoma* spp. | Praziquantel | 0/8 (0%) |

**Likely appropriate therapy based on UpToDate. Waltham, MA: UpToDate Inc. [https://www.uptodate.com](https://www.uptodate.com/) (Accessed on August 26, 2020.)
